# Zooanthroponotic Transmission of Drug-Resistant *Pseudomonas aeruginosa*, Brazil

**DOI:** 10.3201/eid2406.180335

**Published:** 2018-06

**Authors:** Miriam R. Fernandes, Fábio P. Sellera, Quézia Moura, Marcelo P.N. Carvalho, Paula N. Rosato, Louise Cerdeira, Nilton Lincopan

**Affiliations:** Universidade de São Paulo, São Paulo, Brazil (M.R. Fernandes, F.P. Sellera, Q. Moura, M.P.N. Carvalho, L. Cerdeira, N. Lincopan);; Centro Universitário Monte Serrat, Santos, São Paulo (P.N. Rosato)

**Keywords:** reverse zoonosis, zooanthroponosis, nosocomial bacteria, metallo-β-lactamase, household transmission, domicile setting, *Pseudomonas aeruginosa*, bacteria, Brazil, antimicrobial resistance

## Abstract

We recovered VIM-2 carbapenemase-producing *Pseudomonas aeruginosa* isolates from an infected dog, its owner, and the domestic environment. Genomic investigation revealed household transmission of the high-risk hospital clone sequence type 233 in the human–animal–environment interface. Results suggest zooanthroponotic transmission of VIM-2–producing *P. aeruginosa* in the household following the patient's hospital discharge.

The One Health approach has gained worldwide recognition as a valuable way to address critical public health issues, including the problem of antimicrobial drug resistance at the human–animal–environment interface. Although numerous studies have provided substantial evidence of spread of antimicrobial drug–resistant bacteria from animals to humans, current investigations indicate that humans can transmit resistant pathogens to animals in a reverse zoonotic event, called zooanthroponosis (*1**,**2*). Therefore, epidemiologic studies are needed to provide a better understanding of the dynamics of antimicrobial drug resistance transmission between animals and humans. In this study, we investigated an international hospital-associated clone of carbapenem-resistant *Pseudomonas aeruginosa* sequence type (ST) 233 circulating in the human–animal–environment interface of a household setting.

In December 2016, a 5-year-old male Lhasa apso dog was admitted to a veterinary clinic for treatment of head shaking and right ear pruritus. Severe ear canal inflammation and malodorous purulent discharge were observed during clinical examination. A carbapenem-resistant *P. aeruginosa* isolate was recovered from the infected ear (Table). A detailed account of the medical history revealed that the pet owner, a 50-year-old man, had a recent history of hospitalization (of ≈5 months’ duration) for severe traumatic brain injury from a traffic accident, including a 1-month stay in the intensive care unit because of a brain infection, which was treated successfully with vancomycin. The patient was discharged from the hospital 1 month before the onset of infection in the dog.

**Table:** Characteristics of carbapenem-resistant VIM-2 metallo-β-lactamase–producing *Pseudomonas aeruginosa* isolates in a human–animal–environment interface in a household setting, Brazil*

Characteristic	Isolate†		
	ICBDVIM-2	ICBHVIM-2	ICBSVIM-2
Host/environment	Dog	Human	Household environment
Sample	Ear secretion, rectal swab, oral swab	Feces	Sofa swab
Isolation date	2016 Dec 15; 2017 Mar 6	2017 Mar 6	2017 Mar 6
Resistance profile	AMK, AMC, CAZ, CFO, CIP, CL, CPM, CRO, CTX, GEN, IMP, MER, NAL, PPT, STX, TET, TIC	AMK, AMC, CAZ, CFO, CIP, CL, CPM, CRO, CTX, GEN, IMP, MER, NAL, PPT, STX, TET, TIC	AMK, AMC, CAZ, CFO, CIP, CL, CPM, CRO, CTX, GEN, IMP, MER, NAL, PPT, STX, TET, TIC
Carbapenem MIC, μg/mL‡	>32	>32	>32
Resistance genes to:			
β-Lactams	*bla*_VIM-2_, *bla*_PAO_*, bla*_OXA-4_, *bla*_OXA-50_	*bla*_VIM-2_, *bla*_PAO_, *bla*_OXA-4_, *bla*_OXA-50_	*bla*_VIM-2_, *bla*_PAO_, *bla*_OXA-4_, *bla*_OXA-50_
Aminoglycosides	*aadA2, aac(* *3* *)-Id, aph(* *3* *)-IIb*	*aadA2, aac(* *3* *)-Id, aph(* *3* *)-IIb*	*aadA2, aac(* *3* *)-Id, aph(* *3* *)-IIb*
Chloramphenicol	*catB7*, *cmlA1*	*catB7*, *cmlA1*	*catB7*, *cmlA1*
Sulfonamides	*sul1*	*sul1*	*sul1*
Trimethoprim	*dfrB5*	*dfrB5*	*dfrB5*
Tetracyclines	*tetG*	*tetG*	*tetG*
Fosfomycin	*fosA*	*fosA*	*fosA*
Location of *bla*_VIM-2_	Chromosome	Chromosome	Chromosome
MLST,(ST/CC	233/233	233/233	233/233

*AMK, amikacin; AMC, amoxicillin/clavulanic acid; CAZ, ceftazidime; CC, clonal complex; CFO, cefoxitin; CIP, ciprofloxacin; CL, chloramphenicol; CPM, cefepime; CRO, ceftriaxone; CTX, cefotaxime; GEN, gentamicin; IMP, imipenem; MER, meropenem; MLST, multilocus sequence typing; NAL, nalidixic acid; PPT, piperacillin/tazobactam; ST, sequence type; SXT, trimethoprim/sulfamethoxazole; TET, tetracycline, TIC, ticarcillin. †Clonally related *P. aeruginosa* strains ICBDVIM-2 (ear secretion), ICBRVIM-2 (rectal swab), and ICBBVIM-2 (oral swab) were isolated from samples collected in the infected dog. ICBDVIM-2 was isolated on December 15, 2016. ICBRVIM-2 and ICBBVIM-2 were isolated on March 6, 2017. All *P. aeruginosa* strains from the infected dog displayed identical resistance profiles and genetic backgrounds. ‡Imipenem and meropenem.

We conducted an epidemiologic investigation to establish the dynamic of carbapenem-resistant *P. aeruginosa* isolates in the household setting. The household consisted of a married couple without children and owning 2 dogs. We collected surveillance cultures from the owners (fecal samples, n = 2), healthy and infected dogs (rectal and oral cavity, n = 4), and different household sites (sofa, n = 1; living room, n = 2; kitchen, n = 2; bathrooms, n = 3; bedrooms, n = 2; balcony, n = 1; and water cooler, n = 1). We recovered 6 carbapenem-resistant *P. aeruginosa* isolates from the infected dog (rectal and oral cavity), pet owner (fecal samples), sofa, balcony, and water cooler.

We performed whole-genome sequencing of the *P. aeruginosa* isolates (Table) using an Illumina NextSeq platform (Illumina, San Diego, CA, USA). We identified antimicrobial drug resistance genes and multilocus sequence typing of *P. aeruginosa* strains using bioinformatic tools, available from the Center for Genomic Epidemiology (http://genomicepidemiology.org/). We found that all carbapenem-resistant *P. aeruginosa* isolates were clonally related to the hospital-associated lineage ST233, which has been reported as an international high-risk clone, frequently associated with carbapenemase production, and exhibiting resistance to all antimicrobial drugs (*3**–**5*).

In all *P. aeruginosa* strains, carbapenem resistance was associated with the production of VIM-2 metallo-β-lactamase, which was previously reported among clinical *P. aeruginosa* clustered into ST233/clonal complex (CC) 233 in countries in Europe, North America, and Africa, restricted thus far to human nosocomial infections (*3**–**5*).

Genomic data confirmed the household dissemination of VIM-2–producing *P. aeruginosa* ST233 and intestinal colonization of the human host (who had a recent history of hospitalization with a stay in an intensive care unit), suggesting a zooanthroponotic transmission of this nosocomial-adapted clone after the patient’s hospital discharge. A limitation of this study is the lack of data supporting previous episodes of colonization or infection of the pet owner by the VIM-2–producing *P. aeruginosa* during the hospital stay. However, VIM-2–producing *P. aeruginosa* lineages, including ST233, have generally been restricted to human hospital settings (*3**–**6*). In this regard, several studies in hospitalized patients have shown that intestinal colonization with gram-negative bacteria (including carbapenemase producers) persists for >3 months after discharge from the hospital, whereas long-term carriage of >3 years is possible (*7*,*8*). Thus, patients can acquire clinically significant antimicrobial drug–resistant bacteria during hospitalization. As a result, patients harboring these bacteria might transmit them after discharge, mainly to household contacts (*7*,*8*).

In veterinary medicine, the occurrence of VIM-type metallo-β-lactamase–producing *P. aeruginosa* has been restricted to a report of livestock colonization (*9*). We report the further occurrence of VIM-2–producing *P. aeruginosa* in an infected companion animal, showing the emergence of carbapenem-resistant metallo-β-lactamase–producing *P. aeruginosa* in small animal medical care. In this regard, the success of a human hospital-associated lineage of *P. aeruginosa* in animal hosts could be favored by the versatility and adaptation of this opportunistic pathogen, which can survive for long periods in the environment (*10*).

These findings suggest that human hospital-acquired pathogens can colonize and infect companion animals, thus becoming further disseminated in the household environment. Transmission to companion animals could occur not only directly from owners to pets but also from humans to the household environment and then to pets. Because human–pet bonds in household settings could become a critical issue for the transmission of clinically significant multidrug-resistant bacteria, human and veterinary medicine professionals should implement collaborative efforts and health cooperation programs to monitor the spread of such pathogens in the human–animal interface.
